# *In vitro *antimicrobial activity of ten medicinal plants against clinical isolates of oral cancer cases

**DOI:** 10.1186/1476-0711-10-21

**Published:** 2011-05-20

**Authors:** Manju Panghal, Vivek Kaushal, Jaya P Yadav

**Affiliations:** 1 Department of Genetics, M. D. University, Rohtak-124001, Haryana, India; 2 Department of Radiotherapy, Regional Cancer Institute, Pt. B.D.S. Health University, Rohtak-124001, Haryana, India

## Abstract

**Background:**

Suppression of immune system in treated cancer patients may lead to secondary infections that obviate the need of antibiotics. In the present study, an attempt was made to understand the occurrence of secondary infections in immuno-suppressed patients along with herbal control of these infections with the following objectives to: (a) isolate the microbial species from the treated oral cancer patients along with the estimation of absolute neutrophile counts of patients (b) assess the in vitro antimicrobial activity medicinal plants against the above clinical isolates.

**Methods:**

Blood and oral swab cultures were taken from 40 oral cancer patients undergoing treatment in the radiotherapy unit of Regional Cancer Institute, Pt. B.D.S. Health University,

Rohtak, Haryana. Clinical isolates were identified by following general microbiological, staining and biochemical methods. The absolute neutrophile counts were done by following the standard methods. The medicinal plants selected for antimicrobial activity analysis were *Asphodelus tenuifolius Cav., Asparagus racemosus *Willd., *Balanites aegyptiaca *L.*, Cestrum diurnum *L., *Cordia dichotoma *G. Forst, *Eclipta alba *L., *Murraya koenigii *(L.) Spreng. *, Pedalium murex *L.*, Ricinus communis *L. and *Trigonella foenum graecum *L. The antimicrobial efficacy of medicinal plants was evaluated by modified Kirby-Bauer disc diffusion method. MIC and MFC were investigated by serial two fold microbroth dilution method.

**Results:**

Prevalent bacterial pathogens isolated were *Staphylococcus aureus *(23.2%), *Escherichia coli *(15.62%), *Staphylococcus epidermidis *(12.5%), *Pseudomonas aeruginosa *(9.37%), *Klebsiella pneumonia *(7.81%), *Proteus mirabilis *(3.6%), *Proteus vulgaris *(4.2%) and the fungal pathogens were *Candida albicans *(14.6%), *Aspergillus fumigatus *(9.37%). Out of 40 cases, 35 (87.5%) were observed as neutropenic. Eight medicinal plants (*A. tenuifolius, A. racemosus, B. aegyptiaca, E. alba, M. koenigii, P. murex R. communis *and *T. foenum graecum*) showed significant antimicrobial activity (P < .05) against most of the isolates. The MIC and MFC values were ranged from 31 to 500 μg/ml. *P. aeruginosa *was observed highest susceptible bacteria (46.6%) on the basis of susceptible index.

**Conclusion:**

It can be concluded that treated oral cancer patients were neutropenic and prone to secondary infection of microbes. The medicinal plant can prove as effective antimicrobial agent to check the secondary infections in treated cancer patients.

## Background

Over the past decade, advances in the cancer treatment field have been counterbalanced by a rising number of immunosuppressed patients with a multitude of new risk factors for infection [1]. Cancer patients may be immunocompromised due to multiple factors such as chemotherapy, radiotherapy, impairment of normal leukocyte function, or use of corticosteroids [2]. As well as the infections complications remain an important cause of mortality and morbidity between these febrile neutropenic cancer cases [3,4]. They are highly susceptible to almost any type of infection, especially bacterial and fungal infection [5].

Same side the heavy antimicrobial usage particularly in high-risk cancer patients create selection pressures that lead to the emergence of resistant micro-organisms [6]. For instance, many cancer centers have reported an increase in quinolone resistant bacteria (primarily *Escherichia coli *and *Pseudomonas aeruginosa*) in patients receiving quinolone prophylaxis [6-8]. The use of very broad spectrum agents such as the carbapenems has been associated with the selection of multidrug-resistant (MDR) in *Stenotrophomonas maltophilia *and *Pseudomonas aeruginosa *[9-11]. Therefore the increase in antibiotic resistance activity is posing an ever increasing therapeutic problem in cancer case and the ways by which bacteria overcome drug action are many and varied, ranging from intrinsic impermeability to acquired resistance [12-14]. In light of the evidence of rapid spread of resistant clinical isolates, the need to find new antimicrobial agent is of paramount importance for the treatment of infectious diseases in cancer cases.

Researchers are increasingly turning their attention to the medicinal plants and it is estimated that, plant materials are present in, or have provided the models for 25- 50% Western drugs [15,16]. Many commercially proven drugs used in modern medicine was initially used in crude form in traditional or folk healing practices, or for other purposes that suggested potentially useful biological activity. Because the primary benefits of using plant derived medicines are that they are relatively safer than synthetic alternatives, offering profound therapeutic benefits and more affordable treatment [17]. As well as the scarcity of infective disease in wild plants is in itself an indication of the successful defense mechanism developed by them. The defenses mechanisms of the plants may be due to production of enormous variety of small molecules generally classified as 'Phytoalexins'. Phytoalexins are "low molecular weight (MW < 500), anti-microbial compounds that are both synthesized and accumulated in plants after exposure to microorganisms or abiotic agents". Phytoalexins structural spaces contain terpenoids, glycosteroids, flavonoids and polyphenols and these biologically active compounds supposed to exhibit antimicrobial properties [18-20].

Since time immemorial, the traditional medicinal practices have been known for the treatment of various ailments in India. The medicinal plants were selected in this study based on their common uses in Indian traditional systems of medicine [21-27]. Keeping in view, the infectious diseases in oral cancer patients it was important to investigate whether the Indian indigenous plants have any antimicrobial activity against clinical isolates of oral cancer cases. So the present investigation evaluates the infection in immunocompromised oral cancer cases and *in vitro *antimicrobial activity of crude extracts of ten medicinal plants (*Asphodelus tenuifolius Cav., Asparagus racemosus *Willd.*, Balanites aegyptiaca *L.*, Cestrum diurnum *L.*, Cordia dichotoma *G. Forst, *Eclipta alba *L.*, Murraya koenigii *(L.) Spreng.*, Pedalium murex *L.*, Ricinus communis *L. and *Trigonella foenum graecum *L.) against these clinical isolates.

## Materials and methods

Blood and oral swab cultures were taken from 40 oral cancer patients undergoing treatment in the radiotherapy unit of Regional Cancer Institute, Pt. B.D.S. Health University, Rohtak, Haryana (September 2008 to September 2009). These patients were under different treatment protocol i.e., radiotherapy, chemotherapy and radio-chemotherapy both together. The present study was approved by Institutional Human Ethics Committee of the university (M.D. University, Rohtak) and written consent was also taken from the patients. The blood samples (5 ml) were collected from central venous catheter and peripheral vein of the patients at the onset of fever (before the prescription of antibiotics). Blood samples were then transferred in culture bottles of brain heart infusion broth (Hi Media, Mumbai, India). Bottles were incubated at 36.7°C for 7 days. Bottles showing positive growth index were Gram stained and sub cultured on sheep blood agar, sabourad dextrose agar, MacConkey agar and on nutrient agar plates all media were taken from Hi Media, Mumbai, India. These plates were aerobically incubated for 24-48 h at 37°C for isolation of bacterial pathogens and for 24-72 h at 30°C in B.O.D. incubator for fungal pathogenic species isolation. Oral swab were taken by gently rubbing a sterile cotton swab over the labial mucosa, tongue and cancerous lesion [28]. Oral swab were taken at the onset of fever (before the prescription of antibiotics). The swabs were incubated in sheep blood agar, saboured dextrose agar, macconkey agar, nutrient agar, and other selective media for primary isolation of the pathogens. These plates were then aerobically incubated for 24-48 h at 37°C for bacterial pathogens isolation and for 24-72 h at 30°C in B.O.D. incubator for fungal species isolation. When growth was appeared on sub cultured plates of blood and oral swab, all bacterial pathogens were identified by standard microbiological and biochemical procedures. These biochemical tests include carbohydrates fermentation test, urease test, oxidase test, haemolysis of blood, catalase test, motility test and growth of pathogens on specific media etc. [29-32]. A preliminary examination of fungal colony on SDA was done through Gram-stained smear, formation of germ tube, study of micro morphology, morphology on KOH stained smear, assimilation of carbon and nitrogen [29-32]. All isolated pathogens were compared with MTCC standard strains like *S. aureus *with MTCC 96 strain, *S. epidermidis *MTCC 435 strain, *P. vulgaris *MTCC 426 strain, *P. mirabilis *MTCC 425 strain, *E. coli *MTCC 443 strain, *K. pneumonia *MTCC 109 strain, *P. aeruginosa *MTCC 741 strain, *C. albicans *3017 strain and *A. fumigatus *2550 strain respectively.

### Absolute neutrophile count

The absolute neutrophile count (ANC) was done by multiplying the total WBC count by percentage of neutrophils (segmented + band) [5].

Neutropenia: When ANC was less than 500 (sever risk of infection).

Non neutropenia: When ANC was more than 500 (moderate risk of infection).

### Collection of Plant Material

The fruits of *A. tenuifolius*, fresh tubers of *A. racemosus*, mature fruits of *B. aegyptiaca*, aerial part of *C. diurnum*, mature fruits of *C. dichotoma*, whole plant of *E. alba*, mature fruits *P. murex*, leaves of *M. koenigii*, seeds of *R. communis *and leaves of *T. foenum graecum *were collected (October 2008 to December 2009) from different regions of Haryana. The collected plants were identified in the laboratory. Comparison of flora was also made according to different references concerning with the medicinal plants of Haryana and adjoining areas [33-36]. The voucher specimens were deposited in the herbarium of Genetics Department, M. D. University, Rohtak.

All plant parts were dried at room temperature. The dried parts were pounded to fine powder and extracted in different solvents according to their increasing polarity (Petroleum ether, benzene, chloroform, acetone, methanol and aqueous) for 24 h in Soxhlet apparatus. The solvents were recovered with the help of rotary evaporator at 40°C. The quantity of obtained dried plant material varies from 100 gm to 500 gm. The dried extracts were lyophilized in lypholyser. The lyophilized extracts were stored in air tight tubes at 4°C for further anti-microbial analysis.

### Assay for antimicrobial testing

Isolated test bacteria were grown overnight on nutrient agar plates and fungi were grown on sabouraud dextrose agar plates. Bacterial inoculums were prepared from overnight grown cultures (24 h) in peptone water (Hi Media, Mumbai, India), and the turbidity was adjusted equivalent to 0.5 McFarland units (approximately 10^8 ^CFU/ml for bacteria and fungi inoculums turbidity was equivalent at 10^5 ^or 10^6 ^CFU/ml). The microorganisms were inoculated into peptone water and incubated at 35 ± 2°C for 4 h. The positive control was taken streptomycin (10 μg/ml) for antibacterial activity and ketocanozole (10 μg/ml) for antifungal activity. The DMSO added disc was taken as negative control to determine possible inhibitory activity of the dilutant of extract. The susceptibilities of the isolated pathogens were determined by the modified Kirby-Bauer disc diffusion method with Muller Hinton agar plates [37]. Aliquots of inoculums were spread over the surface of agar plates with a sterile glass spreader. To test the antimicrobial activity all extracts were dissolved in DMSO to make a final concentration of 200 mg/ml. 20 μl of each extract was soaked separately into sterile discs (Hi Media, Mumbai, India), and the discs were dried in oven for 4 hours at 35°C. These discs were placed on Muller Hinton agar plates, previously swabbed with the bacterial and fungal inoculum. These plates were incubated for a period of 24 h at 37°C in incubator for bacteria and at 30°C for 24-48 h in B.O.D incubator for fungi. Each experiment was done in triplicate and mean values were taken. Antimicrobial activity was measured in the diameter (mm) of the clear inhibitory zone formed around the disc.

### Minimum inhibitory concentration (MIC) and Minimum fungicidal concentration (MFC)

The MIC and MFC values of extracts were determined based on a microbroth dilution method in 96 multi-well microtitre plates with slight modifications [38]. The crude plant extracts were first diluted to the highest concentration, 40000 to 625 μg/ml), to be tested, and 50 μl of normal saline was distributed from the second to the ninth well. A volume of 50 μl from each extracts was pipetted into the first test well of each microtitre line which acts as sterility control, and then 50 μl of scalar dilution of plant extract was transferred from the second to the ninth well. To each well was added 10 μl of resazurin indicator solution (prepared by dissolving a 270 mg tablet in 40 ml of sterile distilled water). Using a pipette 30 μl of Muller Hinton broth was added to each well to ensure that the final volume was of single strength of the normal saline. Finally, 10 μl of the bacterial suspensions were added to each well. In each plate, a column with a broad-spectrum antibiotic was used as the positive control (streptomycin in serial dilution 40000 to 625 μg/ml).

The plates were wrapped loosely with cling film to ensure that bacterium did not become dehydrated, and were prepared in triplicate. Subsequently, they were placed in an incubator at 37°C for 24 h. Any color change from purple to pink or to colorless was recorded as positive.

The lowest concentration at which the color change occurred was taken as the MIC and MFC value. The average of three values was calculated to determine the MIC and MFC of the test material.

### Preliminary phytochemical analysis

The qualitative preliminary phytochemical analyses of the crude extracts of all plants part isolated from different solvents were performed by following standard methods [39,40].

### Statistical analysis

Each parameter was tested in triplicate. The values were expressed as mean ± standard deviation (SD). The Dunnett one way analysis (ANOVA) was used to determine the significant differences among all columns against control and the P value < .05 was considered as significant. All statistical analysis was performed using Graph Pad Prism version 5.03 software.

### Quantitative evaluation of antimicrobial activity

% of extractive value:

Percent activity: The percent activity demonstrates the total anti- microbial potency of particular extract. It shows number of microbes found susceptible to one particular extract [41].

Bacterial susceptible index (BSI): BSI is used to compare the relative susceptibility between bacterial strains. BSI valves ranges from 0 (resistance to all samples) to 100 (susceptible to all extract [41].

## Results

In the present study the predominant bacterial pathogens isolated were *Staphylococcus aureus *(23.2%), *Escherichia coli *(15.62%), *Staphylococcus epidermidis *(12.5%), *Pseudomonas aeruginosa *(9.37%), *Klebsiella pneumonia *(7.81%), *Proteus mirabilis *(3.6%), *Proteus vulgaris *(4.2%) and the fungal pathogens were *Candida albicans *(14.6%), *Aspergillus fumigatus *(9.37%). On the basis of absolute neutrophile count it was observed that out of 40 cases, 35 (87.5%) were neutropenic. The extracts of ten medicinal plants were prepared in the petroleum ether, benzene, chloroform, acetone, methanol, water and were screened against nine isolates (7 bacterial and 2 fungal sp.). The details of the plants along with their voucher number, family name, common name, parts used, traditional uses and literature source have been listed in Table 1. The phytochemicals isolated from ten medicinal plants were containing alkaloids, amino acids, anthraquinones, cardiac glycosides, flavonoids, reducing sugars, resins, saponins, steroids, terpenes, tannins, proteins, phenols and steroids as shown in Table 1.

**Table 1:** The summary of medicinal plants and their traditional uses

Botanical name (Voucher number)/Local name	Family	Traditional uses	Organ tested	Isolated phytochemicals	Literature Source
*Asphodelus tenuifolius* *Cav*. (MDU 6807)/Piyazi	Liliaceae	The seeds are generally taken for colds and hemorrhoids, a febrifuge and also used for rheumatic pain. Seeds are also used as diuretic agent, healing wound and they are applied externally to ulcers and for inflamed parts.	FR	AL, AN, RS, SA, TA, ST	[21-23]
*Asparagus racemosus *Willd. (MDU 6806)/Shatavari	Liliaceae	The root of the plant is used to promote milk secretion and as demulcent, diuretic, aphrodisiac, antiseptic antiparasitic, antitumor and ant diarrhea. It is also used to treat debility, (especially in women), infertility, impotence, menopause problems, for stomach ulcers, hyperacidity, dehydration, cough and chronic fevers.	TU	AL, SA, TA, FL, RS, PH, AA	[25,27]
*Balanites aegyptiaca *L. (MDU 1601)/Hingote	Balanitaceae	The fruit can used to cure mouth ulcer, whooping cough, sleeping sickness and skin diseases. Fruit kernel has been found as mild laxatives, antidote to arrow poison, and also act as Vermifuge.	FR	AL, CG; FL, RS, SA, TA, PH	[21,22,24]
*Cestrum diurnum *L. (MDU 5112)/Din ka raja	Solanaceae	The leaf paste is used in joint pain.	AP	SP, AL, RS, OL	[22]
*Cordia dichotoma *G. Forst (MDU 4801)/ Leswa	Boraginaceae	The fruit of the plant is used as antihelminthic, diuretic, purgative, useful in dry cough, for cure of jaundice, wound purification, mouth ulcer, and increase male potency.	FR	FL, AL, AA, PR	[22,25]
*Eclipta alba *L. ( MDU 3803)/Bhringraj	Asteraceae	The whole plant is used particularly for alopecia, ringworm, as a hair dye, for liver and spleen enlargement, hepatitis, jaundice and as a general tonic. It has been also used for cataract, cough, hemorrhage, indigestion, toothache, as an antiseptic and a wound healing agent.	WP	SA, FL, PH, OL, PR, AA, AD	[25,27]
*Murraya koenigii *(L.) Spreng. ( MDU 1505)/Kurry patte	Rutaceae	The leave, bark and the root are used intensively in indigenous medicine from ancient time, as a tonic for stomachache, stimulant and carminative. Its leaves are also used in treatment of piles, headache, stomach ache, influenza, rheumatism, traumatic injury, insect, snake bites, anti-vomiting, curing dysentery and diarrhea. Leaves are stimulant and astringent and are used in the treatment of diarrhea, dysentery and diseases of teeth and gum, useful against rheumatism, coughs and hysteria.	LE	AL, RS, SA TA, TR, PR	[21,23,26]
*Pedalium murex *L. (MDU 5401)/Bada Gohkru	Pedaliaceae	The fruit is used as an aphrodisiac, antiseptic, demulcent, diuretic and its decoction is also given in incontinence, spermatorrhoea, nocturnal emission and to promote lochial discharges.	FR	SP, AL, FL, TA, ST, OL, RS, RE	[23,26]
*Ricinus communis *L. (MDU 6210)/Arand	Euphorbiaceae	The extract of seeds is used internally for acute constipation, intestinal inflammation, for removal of worms, rheumatism and as form of birth control. This plant is used as an antidote, bactericide, expectorant, insecticide, larvicidal, laxative, purgative, tonic, and Vermifuge. Castor or castor oil is used as ingredient in folk remedies for abscess, arthritis, asthma, boils, burns, cancer, cold, colic, convulsions, corns, dermatitis, dog bite etc.	FR	AL, FL, PR, ST, OL, RE	[21,24,25]
*Trigonella foenum graecum *L. (MDU 2418)/Methi	Fabaceae	The leaves as a cooked vegetable, is a recommended household remedy for fever and swelling. It also aids in controlling the cough. Leaves also tunes up the appetite, improves digestion, eliminates gaseous distention and relieves the heaviness and abdominal pain.	LE	AL, CG, FL, RS, SA, TA	[21,26]

**Abbreviations**: FR: fruit; LE: leaves; AL: alkaloid; TA: tannins; FA: fatty acids; PR: proteins; FL: flavonoids; OL: oil; RS: reducing sugars; FL: flavonoids; RE: resins; AD: aldehyde; SP: saponins; ST: steroids; AA: amino acids; PH: phenols; AN: anthraquinones; ST: steroids; TR: terpenes; CG: cardiac glycosides.

In the present study the percentage extractive values were ranged from .05 to 17%. The % extractive value and yield of extracts was found maximum in methanol and aqueous extract due to their high polarity and easy solubility of secondary metabolites in the methanol and water. The antimicrobial activity of different plant extract has been listed in Table 2.

**Table 2:** The inhibition Zone (mm) of antimicrobial medicinal plants against clinical isolates of oral cancer cases

Plant species	Fractions extract	*S. a*	*S. e*	*P. v*	*P. m*	*E. c*	*K. p*	*P. a*	*C. a*	*A. f*
*A. tenuifolius*	I	0	0	0	0	0	0	0	0	0
	II	0	0	0	10.33 ± 0.5***	0	10.33 ± 0.5**	0	0	10.66 ± 0.5*
	III	0	10.33 ± 0.5**	10.33 ± 0.5***	10.66 ± 0.5**	11.33 ± 0.5**	15.33 ± 1 ^NS^	11 ± 1.7***	0	10.33 ± 1.5*
	IV	10.33 ± 0.5***	10.67 ± 0.5***	0	11 ± 1.7***	13.33 ± 0.5***	10.33 ± 0.5*	15.67 ± 1.1*	10.33 ± 1.5 ^NS^	10 ± 0 ^NS^
	V	0	11.67 ± 0.5**	10.33 ± 0.5**	10 ± 0*	12.33 ± 1.1**	11 ± 0.57*	0	0	0
	VI	0	0	0	0	13.67 ± 0.5***	0	0	0	0
	Control	20.66 ± 0.5	20 ± 1	18.33 ± 1	17.33 ± 0.5	16.33 ± 1.1	15.33 ± 1.5	19.33 ± 1.1	10.33 ± 0.5	11 ± 1
*A. racemosus*	I	0	0	12.33 ± 0.5***	0	0	0	0	16.66 ± 1.5***	0
	II	0	11.66 ± 0.5***	0	13.66 ± 1.5**	9 ± 1***	13.66 ± 1**	12.66 ± 0.5**	10.33 ± 0.5***	0
	III	0	0	0	0	0	11.66 ± 0.5 ^NS^	0	0	0
	IV	13.66 ± 0.5***	12.66 ± 0.5***	14 ± 1.5***	14.33 ± 0.5***	15.33 ± 0.5^NS^	16.33 ± 1.1 **	13.33 ± 1.1**	11.66 ± 0.5***	11.1 ± 1 ^NS^
	V	0	0	0	0	0	0	0	0	10.33 ± 0.5 ^NS^
	VI	0	0	9.33 ± 1.5	0	0	0	0	13.66 ± 0.5***	0
	Control	18.33 ± 0.5	21.33 ± 2.0	19.33 ± 1	18.33 ± 0.5	14.66 ± 1	18.66 ± 0.5	18.66 ± 0.5	23 ± 0.5	11.66 ± 0.5
*B. aegyptiaca*	I	0	18.33 ± 0.5*	11.66 ± 0.5***	0	12.33 ± 0.5***	0	12.33 ± 1.1***	10.33 ± 1.5***	0
	II	11 ± 0.5***	20.33 ± 0.5^NS^	10.33 ± 0.5***	0	0	10.66 ± 0.5***	10 ± 0***	0	0
	III	0	22.33 ± 0.05 ^NS^	12.33 ± 1.5**	0	11.33 ± 0.5***	11 ± 1***	13 ± 1.7***	0	0
	IV	0	0	0	0	0	0	16.66 ± 1.1**	0	0
	V	10.33 ± 0.5***	18.33 ± 0.5 ^NS^	0	0	0	18.66 ± 0.5*	11.66 ± 0.5***	0	0
	VI	10 ± 1***	12.33 ± 0.5**	0	0	10.66 ± 1.1***	12.33 ± 0.5***	10.66 ± 1.5***	0	0
	Control	20.33 ± 1	25.33 ± 0.05	18.66 ± 0.5	19 ± 1	14.66 ± 0.5	25.33 ± 0.57	21 ± 1.5	18 ± 1	13.33 ± 0.5
*C. diurnum*	I	0	0	0	0	0	0	0	0	0
	II	0	0	0	0	0	0	0	0	0
	III	0	0	0	0	0	0	0	0	0
	IV	0	0	0	0	0	0	0	0	0
	V	0	0	0	0	0	0	0	0	0
	VI	0	10.66 ± 0.5**	0	0	0	0	0	0	0
	Control	21.33 ± 1.5	18.66 ± 1.5	19.33 ± 1.5	20 ± 0.5	15.66 ± 1.1	20.33 ± 1.5	17.33 ± 2.0	11.33 ± 1.5	10.33 ± 0.5
*C. dichotoma*	I	10 ± 0.5**	0	0	0	0	0	0	0	0
	II	0	0	0	0	0	0	0	0	0
	III	0	0	0	0	0	0	0	0	0
	IV	11.33 ± 0.5**	0	0	0	0	0	0	0	0
	V	0	0	0	0	0	0	0	0	0
	VI	0	10.33 ± 0.5**	0	0	0	0	0	0	0
	Control	18.33 ± 0.5	21.33 ± 2.0	19 ± 1	18.33 ± 0.5	15.33 ± 1	18.33 ± 0.05	18.66 ± 0.08	10.33 ± 0.5	11.33 ± 0.5
*E. alba*	I	0	0	0	0	11.66 ± 1.1***	10.33 ± 0.5**	10.33 ± 0.5***	0	0
	II	0	11.33 ± 0.5***	0	10.33 ± 0.5***	11 ± 1***	12.67 ± 0.5*	0	0	0
	III	10.33 ± 0.5***	10.33 ± 0.5***	0	0	0	11.66 ± 0.5*	0	12 ± 0 ^NS^	0
	IV	0	0	0	0	0	12.66 ± 0.5*	0	0	0
	V	0	0	0	0	0	0	10 ± 1**	13.33 ± 1.1 ^NS^	0
	VI	0	0	0	0	0	0	11.33 ± 1.5**	0	0
	Control	18.33 ± 0.05	18.66 ± 1.5	17.33 ± 0.5	17.66 ± 0.5	16.66 ± 1.1	20.33 ± 1.5	17 ± 1.7	11.66 ± 1.5	11.33 ± 1.5
*M. koenigii*	I	12 ± 1***	0	0	0	0	0	10.33 ± 0.5**	0	10.33 ± 0.5 ^NS^
	II	14.33 ± 0.5**	13.33 ± 0.5**	13.67 ± 0.5***	14.33 ± 0.5**	13 ± 0.5*	10.33 ± 0.5**	10.67 ± 0.5***	10.67 ± 0.5 ^NS^	14.33 ± 0.5 ^NS^
	III	0	0	0	0	0	10.33 ± 0.5**	0	0	10.67 ± 0.5 ^NS^
	IV	14.33 ± 0.5**	14.33 ± 0.5*	14.33 ± 0.5***	10.33 ± 0.5***	0	12.67 ± 0.5**	10 ± 0**	10.33 ± 0.5 ^NS^	14.33 ± 0.5 ^NS^
	V	0	0	0	0	0	0	0	0	0
	VI	0	0	11.67 ± 0.5***	0	0	0	0	0	0
	Control	18.66 ± 0.5	19.66 ± 0.5	25.66 ± 1	20.3 ± 0.5	19.66 ± 0.5	15 ± 0.5	19.33 ± 1.1	10.33 ± 0.5	11 ± 1
*P. murex*	I	0	14.33 ± 0.5***	0	0	0	0	0	0	0
	II	0 0	0 0	0 0	0 0	0 0	0 0	0 0	0 0	0 0
	III	14.66 ± 0.5***	10.33 ± 1***	0	0	0	0	0	0	0
	IV	9.66 ± 0.5**	10.66 ± 0.5***	0	0	10.66 ± 1.1***	13.33 ± 1.1***	0	0	0
	VI	0	0	0	0	0	0	10.33 ± 0.5***	9.33 ± 1.5**	0
	V	0	0	0	0	0	0	12 ± 0**	11 ± 1*	8 ± 0.5 ^NS^
	Control	21.33 ± 1.5	25.33 ± 0.5	19.33 ± 1.5	20.33 ± 0.5	19.33 ± 0.5	2.333 ± 2.6	17.33 ± 2.0	12.66 ± 1.1	10.33 ± 0.5
*R. communis*	I	10.66 ± 1.1***	0	0	0	0	0	10.33 ± 0.5***	0	-
	II	12.33 ± 0.5**	0	10.66 ± 0.5***	11.66 ± 0.5***	0	11.33 ± 1.5***	12.33 ± 1.1***	0	-
	III	0	0	0	0	0	0	11 ± 1.7***	0	-
	IV	0	0	10.33 ± 1.5***	0	0	0	12.66 ± 1.5***	0	-
	V	0	0	0	0	12.33 ± 1.1***	10.33 ± 0.5***	10 ± 0***	0	-
	VI	0	9.66 ± 0.5**	0	0	14.33 ± 1.1***	0	16 ± 0.5*	0	-
	Control	20.66 ± 1.5	19.66 ± 0.5	19.66 ± 0.5	17.33 ± 0.5	16.33 ± 0.5	20.33 ± 2.0	19.33 ± 1.1	10.33 ± 0.5	11 ± 1
*T. foenum graecum*	I	11 ± 1.1***	12.33 ± 1 ^NS^	10.67 ± 0.5***	10.33 ± 0.5**	10.33 ± 1.5***	11.33 ± 0.5**	10.33 ± 0.5**	0	13.1 ± 1.1 ^NS^
	II	13.67 ± 1.5**	11.67 ± 0.5***	12.33 ± 0.5**	11.66 ± 0.5***	12.33 ± 0.5***	10.33 ± 0.5**	0	11.67 ± 0.5 ^NS^	0
	III	10.33 ± 0.5**	13.660.5**	12.67 ± 0.5***	0	10.67 ± 0.5**	10.33 ± 0.5***	0	10 ± 0.5 ^NS^	0
	IV	0	0	0	0	0	0	10 ± 0***	0	0
	V	0	0	0	0	0	0	10.33 ± 0.5**	0	0
	VI	0	0	0	0	0	0	11.33 ± 0.5**	0	0
	Control	18.33 ± 0.5	17.33 ± 0.5	26 ± 1	17.33 ± 0.5	18.33 ± 1	16 ± 2	19.33 ± 1.1	10.33 ± 0.5	11 ± 1

**Abbreviations**: 0, no activity; S. a, *S. aureus*; S. e, *S. epidermidis*; P. v, *Proteus vulgaris*; P. m, *Proteus mirabilis*; E. c, *E.coli*; K. p, *Klebsiella pneumonia*; P. a, *Pseudomonas aeruginosa*; C. a, *Candida albicans*; A.s *Aspergillus fumigatus*; Control, Streptomycin (bacteria), Ketocanozole (fungi); I, Petroleum ether; II, Benzene; III, Chloroform; IV, Acetone; V, Methanol; VI Aqueous; P < .05, less significant*; P < .01, significant**; P < .001, highly significant***.

The antimicrobial activity different extracts of *A. tenuifolius, A. racemosus, B. aegyptiaca, C. diurnum, C. dichotoma, E. alba, P. murex, M. koenigii, R. communis *and *T. foenum graecum *on different bacterial and fungal clinical isolates has been shown Table 2 as mean ± SE. *S. aureus *exhibited good susceptibility to acetone extract of *A. tenuifolius, A. racemosus, C. dichotoma, M. koenigii *and *P. murex. S. epidermidis *exhibited remarkable susceptibility to all solvent extract (except acetone) of *B. aegyptiaca*. Pet. ether, benzene and chloroform extracts of *B. aegyptiaca *and *T. foenum graecum *revealed significant antibacterial activity against *P. vulgaris*. Our study revealed *P. mirabilis *as most resistance bacterial species due very limited activity observed in all plant extracts. Out of six solvents, benzene extract of *A. tenuifolius, A. racemosus, E. alba, P. murex R. communis *and *T. foenum graecum *exhibited good antibacterial activity against *P. mirabilis*. Like *P. mirabilis, E. coli *also found susceptible to benzene extract of *A. racemosus, E. alba, P. murex *and *T. foenum graecum*. The benzene extracts of *A. racemosus, A. tenuifolius, B. aegyptiaca, E. alba, P. murex, R. communis *and *T. foenum graecum *also exhibit very good susceptibility to *K. pneumonia. P. aeruginosa *which is considered as very resistance species in infectious diseases was observed 100% susceptible to all solvent extracts of *R. communis*. Out of selected medicinal plants *A. racemosus *exhibited good antifungal activity against *C. albicans. A. fumigatus *was found resistant to *B. aegyptiaca, C. diurnum, C. dichotoma, E. alba, M. koenigii, R. communis *and *T. foenum graecum *extracts.

In our study the percent activity i.e the total anti- microbial potency of particular extract was evaluated from 14.2 to 100%. The acetone extract of *A. racemosus *and the Pet. ether of *T. foenum graecum *showed 100% activity to all clinical isolates. The present study also revealed that the *P. aeruginosa *was highest susceptible bacteria with 46.6%% susceptibility followed by *K. pneumonia *(40%), *S. epidermidis *(35%), *S. aureus *(30%), *E. coli *(30%), *P. vulgaris *(26.6%) and *P. mirabilis *(20%).

The MIC and MFC of different solvent extracts against clinical isolates has been listed in Table 3. The MIC ranges from 31 to 500 μg/ml and similarly the MFC ranges from 31 to 500 μg/ml. The minimum MIC (31 μg/ml) of all extracts was observed in the following way, the acetone extract of *A. racemosus *against *P. vulgaris *and *K. pneumonia*, the methanolic extract of *B. aegyptiaca *and *R. communis *against *K. pneumonia*, the aqueous extract of *R. communis *against *P. aeruginosa*, aqueous and methanolic extract of *T. foenum- graecum *against *P. aeruginosa*.

**Table 3:** MIC and MFC (μg/ml.) of antimicrobial fractions against clinical isolates of oral cancer cases

Plant species	Fractions extract	S. a	S. e	P. v	*P. m*	*E .c*	*K. p*	*P. a*	*C. a*	*A. f*
*A. tenuifolius*	I	0	0	0	0	0	0	0	0	0
	II	0	0	0	125	0	500	0	0	250
	III	0	250	125	500	250	500	500	0	250
	IV	125	125	0	250	250	250	250	125	500
	V	0	250	250	250	250	250	0	0	0
	VI	0	0	0	0	62.5	0	0	0	0
*A. racemosus*	I	0	0	250	0	0	0	0	125	0
	II	0	500	0	125	250	62.5	250	250	0
	III	0	0	0	0	0	62.5	0	0	0
	IV	125	125	31	125	125	31	250	500	31
	V	0	0	0	0	0	0	0	62.5	500
	VI	0	0	500	0	0	0	0	0	0
*B. aegyptiaca*	I	0	250	250	0	250	0	62.5	125	0
	II	500	125	250	0	0	250	125	0	0
	III	0	62.5	125	0	62.5	250	500	0	0
	IV	0	0	0	0	0	0	125	0	0
	V	250	125	0	0	0	31	62.5	0	0
	VI	250	250	0	0	62.5	125	125	0	0
*C. diurnum*	I'	0	0	0	0	0	0	0	0	0
	II	0	0	0	0	0	0	0	0	0
	III	0	0	0	0	0	0	0	0	0
	IV	0	0	0	0	0	0	0	0	0
	V	0	0	0	0	0	0	0	0	0
	VI	0	125	0	0	0	0	0	0	0
*C. dichotoma*	I	250	0	0	0	0	0	0	0	0
	II	0	0	0	0	0	0	0	0	0
	III	0	0	0	0	0	0	0	0	0
	IV	125	0	0	0	0	0	0	0	0
	V	0	0	0	0	0	0	0	0	0
	VI	0	125	0	0	0	0	0	0	0
*E. alba*	I	0	0	0	0	250	250	250	0	0
	II	0	125	0	125	250	250	0	0	0
	III	250	500	0	0	0	500	0	250	0
	IV	0	0	0	0	0	250	0	0	0
	V	0	0	0	0	0	0	125	250	0
	VI	0	0	0	0	0	0	125	0	0
*M. koenigii*	I	250	0	0	0	0	0	250	0	125
	II	125	250	125	62.5	125	250	250	125	125
	III	0	0	0	0	0	125	0	0	125
	IV	62.5	125	31	125	0	62.5	125	125	250
	V	0	0	0	0	0	0	0	0	0
	VI	0	0	62.5	0	0	0	0	0	0
*P. murex*	I	0	500	0	0	0	0	0	0	0
	II	0	0	0	0	0	0	0	0	0
	III	125	125	0	0	0	0	0	0	0
	IV	500	250	0	0	125	500	250	0	0
	V	0	0	0	0	0	0	125	0	250
	VI	0	0	0	0	0	0	0	0	250
*R. communis*	I	250	0	0	0	0	0	500	0	0
	II	125	0	125	250	0	500	250	0	0
	III	0	0	0	0	0	0	250	0	0
	IV	0	0	250	0	0	0	250	0	0
	V	0	0	0	0	250	31	250	0	0
	VI	0	62.5	0	0	250	0	31	0	0
*T. foenum-graecum*	I	31	250	250	250	125	62.5	125	0	125
	II	62.5	31	125	125	31	31	0	125	0
	III	125	62.5	125	0	250	125	0	125	0
	IV	0	0	0	0	0	0	62.5	0	0
	V	0	0	0	0	0	0	31	0	0
	VI	0	0	0	0	0	0	31	0	0

**Abbreviations**: 0, no activity; S. a: *S. aureus*; S. e: *S. epidermidis*; P. v: *Proteus vulgaris*; P. m: *Proteus mirabilis*; E. c: *E.coli*; K. p: *Klebsiella pneumonia*; P. a: *Pseudomonas aeruginosa*; C. a: *Candida albicans*; A.s: *Aspergillus fumigatus*; Control, Streptomycin (bacteria), Ketocanozole (fungi); I: Petroleum ether; II: Benzene; III: Chloroform; IV: Acetone; V: Methanol; VI: Aqueous.

But on the other hand the minimum MFC (31 μg/ml) was exhibited in acetone extract of *A. racemosus *against *A. fumigatus*. The potency of the extract based on the zones of inhibitions, was compared with standard commercial available antibiotics such as streptomycin (10 μg/ml) and ketacanzole at (10 μg/ml) observed that the antimicrobial activity of crude extracts was less than those of the standard drugs.

## Discussion

Patients with cancer develop serious bacterial and fungal infections often, especially during periods of cancer treatments. Antibiotic usage for the prevention and treatment of bacterial infections in these high-risk patients leads to selection pressures resulting in the emergence and spread of resistant organisms. Many organisms acquire several resistances mechanisms; making them multi-drug-resistant (MDR). The development of novel antimicrobial agents with activity against pathogens that have become resistant to currently available agents is one tactics for combating resistant organisms [42]. Therefore the rapid propagation in antibiotic resistance and the increasing interest in natural products have placed medicinal plants back in the front lights as a reliable source for the discovery of active anti-microbial agents and possibly even novel classes of antibiotics [43]. So in keeping view all the use of medicinal plants our study was based on isolation of microbes of oral cancer patients and antimicrobial activity of medicinal plants against these clinical isolates.

In the present study we found that seeds extract of *A. tenuifolius*, the tuber extracts of *A. racemosus*, fruit extract of *B. aegyptiaca*, leaves extract of *T. foenum- graecum*, showed antibacterial activity against most of the isolated microbes from the oral cancer cases. The significant antifungal activity was exhibited by different extracts of *A. racemosus *(P < .05) against *C. albicans *but in contrast to that no significant antifungal activity was observed against *A. fumigatus*.

Although numerous studies have in the past, focused on antimicrobial activity of *A. racemosus, A. tenuifolius, B. aegyptiaca, E. alba, P. murex, R. communis *and *T. foenum graecum *against various bacteria and fungi [27,44-46] but very few studies have been focused on the antimicrobial activity of these medicinal plants against clinical isolates.

Methanol and water extracts of *A. racemosus *tubers were screened against *E. coli*. S. *aureus, K. pneumonia, P. aeruginosa *and *C. albicans *obtained from R.N.T. Medical College, Udaipur (Rajasthan). Antimicrobial activity was observed against all the studied microbes with inhibition zone range from 7 to 24 mm [47]. On the contrary we have not observed antibacterial activity against *E. coli, S. aureus, K. pneumonia *and *P. aeruginosa*. Another study the showed the antifungal activity of tubers methanol extract against *C. albicans *(16 mm) isolated from vaginal thrush patients, visiting Rajah Muthiah Medical College and Hospital, Annamalai Nagar, Tamilnadu, India [48]. Our study revealed the almost similar antifungal activity of *A. racemosus *tubers against *C. albicans *(13 mm).

The methanol and ethanol leaf extracts of *E. alba *was evaluated for the antimicrobial activity against human isolated pathogens (*E. coil, K. pneumonia, S. aureus *and *P. aeruginosa *) obtained from Department Microbiology, Mysore Medical College and Research Institute, Rajiv Gandhi University, Mysore, India [49]. It was observed that both extracts exhibited antibacterial activity against all bacteria and inhibition zone was observed ranged from 9 to 20 mm. But in contrast to above study we found that methanol and aqueous extract of *E. alba *have no inhibition against *E. coli, K. pneumonia *and *S. aureus *but activity was only found against *P. aeruginosa *(10 mm, 11 mm).

To a large extent, the chronological age of the plant, percentage humidity of the harvested material, situation and time of harvest, solvent used and the method of extraction were possible sources of variation for the bioactivity of the extracts [50-52].

As it is well known that *P. aeruginosa *is notorious for its resistance to antibiotics and is, therefore particularly dangerous and dreaded pathogen. The bacterium is naturally resistance to many antibiotics due to the permeability barriers afforded by its outer membrane composed of lipopolysaccharide (LPS). Also, its tendency to colonize surfaces in a biofilm form makes the cells impervious to therapeutic concentrations of antibiotics. One method to reduce the resistance to antibiotics is by using antibiotics resistance inhibitors from plant origin [53,54]. We have observed very significant (P < .05) antibacterial activity of *B. aegyptiaca *and *R. communis *all extracts, against *P. aeruginosa*. These results revealed that the antibacterial activity of *R. communis *and *B. aegyptiaca *extracts hold the most promise for further work against this bacterium through toxicity testing.

In our study various phytochemicals compounds alkaloids, amino acids, anthraquinones, cardiac glycosides, flavonoids, reducing agents, resins, saponins, terpenes, tannins, proteins, phenols and steroids were isolated from ten medicinal plants. The antimicrobial susceptibility of studied medicinal plants could be attributed to the presence of these antimicrobial compounds, which is also proved by other studies [55-62].

The results of our research highlights, the fact that the organic solvent extracts exhibited greater antimicrobial activity because the antimicrobial principles were either polar or non-polar and they were extracted only through the organic solvent medium [63,64]. So the present observation suggests that the organic solvent extraction was suitable to verify the antimicrobial properties of medicinal plants which are also supported by many other investigators [65-68].

In nut shell, our study justifies the claimed uses of *A. tenuifolius, A. racemosus, B. aegyptiaca, E. alba, P. murex, M. koenigii, R. communis *and *T. foenum graecum *in the Indian traditional system of medicine to treat various diseases. However, as the toxic affects of plant extracts were not explored or tested in present study, so the usefulness of these extracts as antimicrobial compound can only be predicated after toxicity testing on eukaryotic cells.

## Conclusion

The present investigation reported that the secondary infections in the immunocompromised oral cancer cases were due to bacterial and fungal species. This study also report that the medicinal plants (*A. tenuifolius, A. racemosus, B. aegyptiaca, E. alba, P. murex, M. koenigii, R. communis *and *T. foenum graecum*) used in Indian-herbal medicine may possess antimicrobial activity against the clinical isolates of oral cancer cases. These findings can form the basis for further studies to toxicity testing, isolate active compounds, elucidate the structures, and also evaluate them against wider range of resistance bacterial and fungal strains of cancer patients with the goal to find new therapeutic principles.

## Competing interests

The authors declare that they have no competing interests.

## Authors' contributions

All authors have equal contribution in study designs, experiment, data analysis and interpretation of data. Similarly all authors have critically reviewed the manuscript, approved of its contents and consented to its communication for publication
